# Development and approval of a Lasso score based on nutritional and inflammatory parameters to predict prognosis in patients with glioma

**DOI:** 10.3389/fonc.2025.1280395

**Published:** 2025-01-23

**Authors:** Huixian Li, Hui Hong, Jinling Zhang

**Affiliations:** ^1^ The Second Medical College, Binzhou Medical University, Binzhou, Shandong, China; ^2^ JGraduate School of Jinzhou Medical University, jinzhou, China; ^3^ Department of Oncology, Linyi People's Hospital, Linyi, China

**Keywords:** glioma, nutrition indices, inflammation indices, Lasso score, prognosis

## Abstract

**Objectives:**

Preoperative peripheral hematological indices, including the neutrophil-to-lymphocyte ratio (NLR), platelet-to-lymphocyte ratio (PLR), monocyte-to-lymphocyte ratio (MLR), and prognostic nutritional index (PNI), exhibit promise as prognostic markers for glioma. This study evaluated the prognostic value of a combined scoring system incorporating NLR, PLR, MLR, and PNI, and developed a nomogram to predict glioma prognosis.

**Methods:**

Data on preoperative NLR, PLR, MLR, and PNI were collected from 380 patients with pathologically diagnosed glioma (266 in the training cohort, 114 in the validation cohort). The Least Absolute Shrinkage and Selection Operator (Lasso) was employed to select relevant hematological indicators and generate a Lasso score. A nomogram was constructed utilizing Cox regression and Lasso variable selection. This nomogram incorporated the Lasso score, age, pathological type, chemotherapy status, and Ki67 expression to predict overall survival (OS). Model performance was evaluated utilizing Harrell’s c-index, calibration curves, DCA, and clinical utility (stratification into low-risk and high-risk groups), and verified utilizing the independent validation cohort.

**Results:**

A total of 380 glioma patients were enrolled and separated into training (n = 266) and validation (n = 114) cohorts. The two cohorts demonstrated no significant differences in baseline characteristics. NLR, PLR, MLR, and PNI from the training dataset were utilized for Lasso calculation. Multivariable analysis indicated that age, pathological grade, chemotherapy status, Ki-67 expression, and the Lasso score were independent predictors of OS and were then included in the nomogram. The nomogram model based on the training cohort had a C index of 0.742 (95% CI: 0.700-0.783) and AUC values of 0.802, 0.775, and 0.815 for ROC curves at 1, 3, and 5 years after surgery. The validation cohort derived a similar C-index of 0.734 (95% CI: 0.671–0.798) and AUC values of 0.785, 0.778, and 0.767 at 1, 3, and 5 years, respectively. The nomogram demonstrated good calibration in both cohorts, indicating strong agreement between predicted and observed outcomes. The threshold probabilities for DCA at 1-, 3-, and 5-years post-surgery in the training and validation cohorts were 0.08~k0.74, 0.25~0.80, and 0.08~0.89, and 0.13~0.60, 0.28~0.81, and 0.25~0.88, respectively.

**Conclusions:**

A nomogram incorporating a Lasso score effectively predicted prognosis in glioma patients. However, its performance did not significantly exceed that of standard clinical nomograms.

## 1 Introduction

Gliomas constitute the most prevalent intracranial tumors in the central nervous system. Originating primarily from glial cells, these tumors exhibit aggressive behavior, represented by insufficiently defined boundaries and poor prognosis (1). The 2021 World Health Organization CNS5 classification system for tumor types categorizes gliomas into four principal groups: human-type diffuse gliomas, pediatric-type diffuse low-grade gliomas, pediatric-type diffuse high-grade gliomas, and circumscribed astrocytomas. Lower-grade gliomas, including diffuse astrocytic glioma and oligodendroglioma, typically are in WHO CNS5 grades 1–2. Higher-grade gliomas are generally assigned grades 3–4, with glioblastoma representing the most frequent diagnosis (2, 3). Prognostic outcomes for glioma patients are largely contingent upon tumor grade; individuals with lower-grade gliomas generally experience a better prognosis; whereas, glioblastomas exhibit the greatest degree of malignancy, comprising 48.3% of all malignant central nervous system tumors, and carry a dismal prognosis, with a 5-year survival rate of mere 6.8% (4).

Notwithstanding advances in primary treatment modalities for gliomas, including neurosurgery, radiotherapy, chemotherapy, and targeted therapy, patient prognosis remains poor. The highly invasive characteristics of high-grade gliomas and the recurrence rate contribute to a median overall survival (OS) of only 12–18 months (5).

Accurate prognostication can facilitate stratified patient management, enabling individualized treatment and follow-up strategies. This personalized approach offers the potential to enhance both patient prognosis and quality of life.

A large body of studies have evaluated the effect of preoperative peripheral hematological indices related to nutrition, coagulation, and inflammation on the clinical prognosis of cancer patients. The neutrophil-to-lymphocyte ratio (NLR) was found to be an independent predictor of OS in a study of 128 glioma patients, with high NLR values indicating a poorer prognosis. In contrast, the prognostic nutritional index (PNI) and platelet-to-lymphocyte ratio (PLR) did not independently predict OS in this cohort (6). However, He et al. demonstrated the predictive utility of PNI in grade IV glioma patients, observing increased clinical treatment benefit among those with higher PNI values (7). Wang et al. analyzed preoperative inflammatory markers in glioblastoma patients and found that both PLR and NLR independently predicted OS, with higher PLR associated with a worse prognosis (8). Sakane et al. evaluated the relationship between peripheral hematological markers of inflammation and nutrition and the prognosis of patients with thymic epithelial tumors after complete resection. Their analysis indicated that the monocyte-to-lymphocyte ratio (MLR) was an independent prognostic factor for disease-free survival (DFS), where a higher MLR correlated with poorer DFS (9). Increasingly, research is expanding beyond simple correlations between tumor prognosis and inflammatory or nutritional indices. Recent studies suggest that integrating clinical features and inflammatory markers can generate robust clinical prediction models. For instance, a nomogram model for predicting glioma patient prognosis was developed by combining tumor resection range, tumor grade, and NLR (10). Other analyses have proposed scoring systems based on multiple peripheral blood inflammatory markers. One such clinical prediction model combined serum albumin and NLR scores and demonstrated strong predictive accuracy for glioblastoma patient outcomes (11).

The prognostic value of preoperative peripheral blood inflammatory markers and related nutritional indices in glioma remains to be elucidated, and analyses into composite scoring systems incorporating multiple inflammatory markers for glioma prognosis prediction are currently absent. A thorough review of the literature indicated no studies utilizing a combination of NLR, PLR, MLR, and PNI scoring systems with relevant clinical indices to construct a clinical prediction model for gliomas.

**Figure 1:**
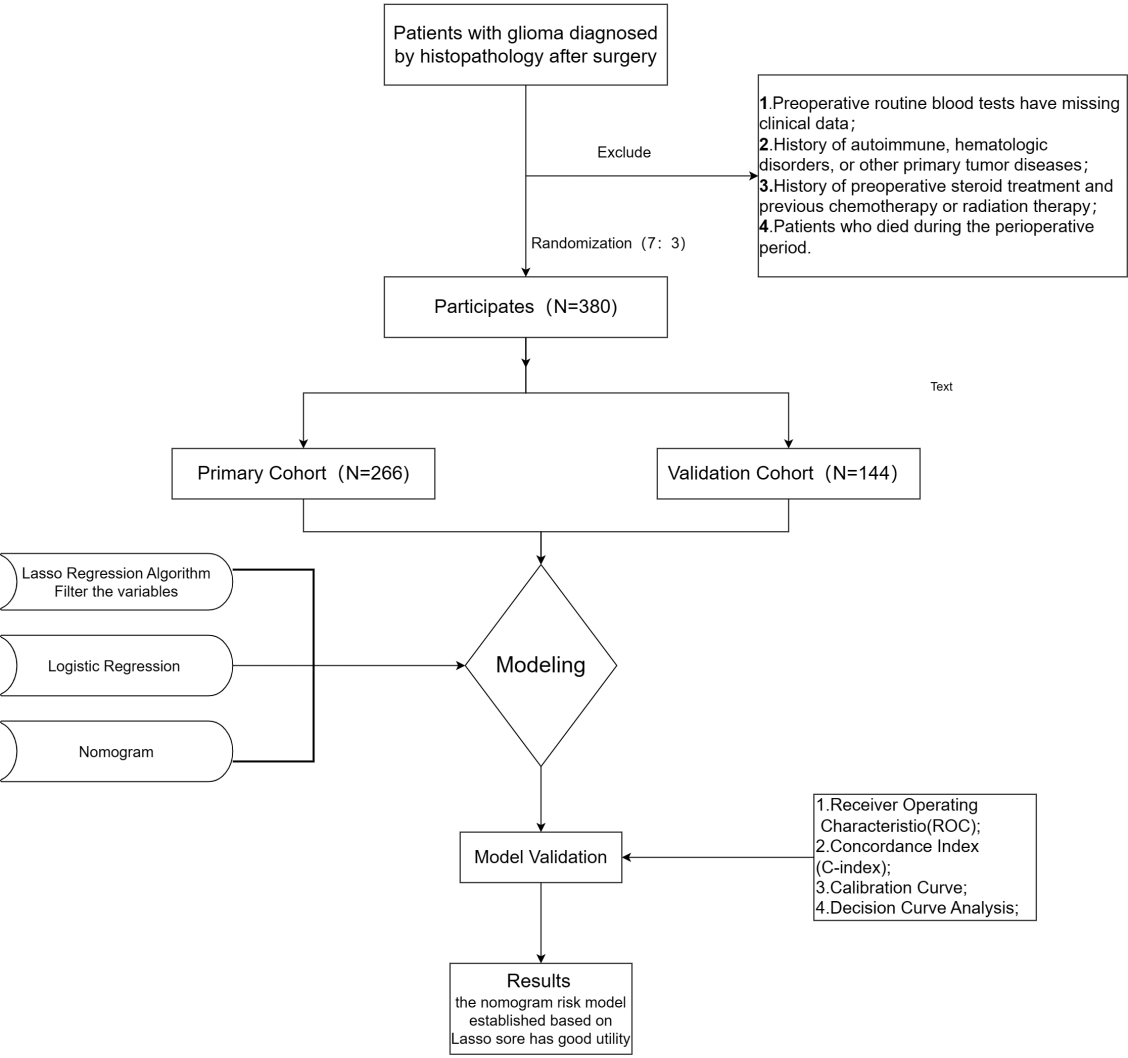
Flow diagram of study design.

This study, therefore, sought to develop a streamlined scoring system integrating the four inflammatory indices of NLR, PLR, MLR, and PNI, and to establish a clinical prediction model for glioma prognosis based on this system in conjunction with relevant clinical characteristics. The study design is illustrated in **Figure 1**.

## 2 Methods

### 2.1 Participants

We conducted a retrospective analysis of clinical data from 380 glioma patients who received surgical resection at Linyi People’s Hospital between April 2013 and September 2021. Patients were categorized into two cohorts: a training cohort (n = 266) and a validation cohort (n = 114). Inclusion criteria were: (1) histopathologically confirmed glioma diagnosis; (2) complete clinical data available from preoperative routine blood tests and liver function tests (including neutrophils, lymphocytes, monocytes, platelets, and albumin); (3) no autoimmune or hematological diseases; (4) no history of other primary malignancies; and (5) no perioperative mortality (approximately 5–7 days before to 7–12 days after surgery). Glioma grading and diagnosis were determined primarily utilizing the WHO classification criteria. This study was approved by the ethics committee of Linyi People’s Hospital, and all participants offered informed consent.

### 2.2 Blood examinations and data collection

Patient demographics and clinicopathological characteristics, including sex, age, smoking and alcohol use history, initial symptoms, extent of resection (“Partial resection” is defined as less than 95% of the preoperative tumor volume removed, while “Complete resection” indicated removal of 95% or more), midline shift, tumor texture and boundaries, radiation and chemotherapy treatments, body mass index (BMI), Karnofsky Performance Scale score (KPS; scored 0-100, with 0 representing death from disease and 100 representing a normal, asymptomatic state), tumor location, pathological classification, isocitrate dehydrogenase status, p53 status, Ki-67 index, methylguanine-DNA methyltransferase status, and glial fibrillary acidic protein status, were collected for all participants. Nutritional indices were calculated utilizing the following formulas: PNI = 10 × albumin (g/dL) + 5 × lymphocyte count (109/L), and BMI = body weight (kg)/height (m)^2^. NLR, PLR, and MLR were utilized inflammation-based indices. Blood samples for routine assessment of neutrophil, lymphocyte, and absolute monocyte counts, as well as serum albumin levels, were collected seven days prior to surgery. OS was defined as the time from the date of glioma resection to either death or the last follow-up.

### 2.3 Statistical analysis

Statistical analyses were performed utilizing R (version 4.2.2). Normally distributed variables were presented as mean ± standard deviation and compared utilizing t-tests. Non-normally distributed variables were presented as median and interquartile range (P25, P75) and compared utilizing rank-sum tests. Categorical variables were described as a percentage of cases (%) and analyzed with χ2 or Fisher’s exact tests. The complete dataset was randomly divided 7:3 into a model-training set (70%) and a model-validation set (30%) utilizing the sample function in R.

A predictive model was developed from the training dataset. Potential predictors of overall survival (OS) were screened utilizing univariate Cox regression analysis, with OS representing the dependent variable (P<0.05). Variables demonstrating non-zero coefficients were then selected through the application of Least Absolute Shrinkage and Selection Operator (Lasso) Cox regression. A 10-fold cross-validation procedure determined the optimal parameter configuration, specifically identifying coefficients based on the lambda value corresponding to one standard error of distance from the minimum deviation. This process also facilitated the removal of variables with zero coefficients. The remaining variables were subjected to multivariable Cox regression analyses to identify independent predictors (P<0.05) for inclusion in the predictive nomogram model. The developed nomogram model was cross-validated, and calibration curves were generated to determine the degree of calibration. The concordance index (C-index) was calculated, and the model’s discriminatory ability was further evaluated through time-dependent receiver operating characteristic (ROC) curve analysis, calculating the area under the curve (AUC) and other relevant indices. Clinical decision curve analysis (DCA) was conducted to evaluate the clinical applicability of the model and quantify the net benefit across the range of threshold probabilities. Finally, external validation of the constructed nomogram model was performed utilizing the validation dataset. In addition, nomogram scores were calculated for all study participants based on the finalized model. Optimal cutoff values for these nomogram scores were determined in the training set utilizing the surv_cutpoint function in the R package survminer. With this cutoff value, all participants were stratified into high- and low-risk groups, and Kaplan-Meier curves were generated and compared through log-rank testing.

The Lasso Cox regression model was implemented utilizing the “glmnet” software package. The R packages “riskRegression”, “ggprism”, “ggplot2”, and “rms” were utilized for nomogram creation and visualization. Clinical decision curves were analyzed employing the “ggDCA” package, and survival analyses were conducted with the “survminer” package. A two-sided p-value of less than 0.05 (P<0.05) was considered significant throughout the study.

## 3 Results

### 3.1 Baseline patient characteristics

This study included 380 patients diagnosed with gliomas. This patient population was divided into a training cohort of 266 patients and a validation cohort of 114 patients. There were no significant differences observed between the training and validation cohorts for any of the recorded variables (P>0.05). The median age of the patients in the overall cohort was 46.50 years. In the training cohort, 154 patients (57.89%) were male, and in the validation cohort, 60 patients (52.63%) were male. Further detailed clinicopathological and demographic information for all patients is presented in **Table 1**.

**Table 1:** Baseline characteristics.

	[ALL]	Training	Validation	p.overall
	*N=380*	*N=266*	*N=114*	
Age	46.50 [36.00;57.00]	47.00 [36.25;56.00]	45.50 [36.25;57.75]	0.843
Gender:				0.404
Men	214 (56.32%)	154 (57.89%)	60 (52.63%)	
Women	166 (43.68%)	112 (42.11%)	54 (47.37%)	
BMI	23.85 [22.03;26.71]	23.88 [22.03;26.76]	23.51 [22.03;26.69]	0.794
Kps	70.00 [60.00;70.00]	70.00 [60.00;70.00]	70.00 [60.00;70.00]	0.462
Surgical removal:				0.641
Complete resection	268 (70.53%)	190 (71.43%)	78 (68.42%)	
Partial resection	112 (29.47%)	76 (28.57%)	36 (31.58%)	
Midline shift:				0.289
No	214 (56.32%)	155 (58.27%)	59 (51.75%)	
Yes	166 (43.68%)	111 (41.73%)	55 (48.25%)	
Tumor texture:				0.541
Hard	105 (27.63%)	70 (26.32%)	35 (30.70%)	
Other	113 (29.74%)	78 (29.32%)	35 (30.70%)	
Soft	162 (42.63%)	118 (44.36%)	44 (38.60%)	
Tumor margin:				0.851
Obscure	357 (93.95%)	249 (93.61%)	108 (94.74%)	
Well-defined	23 (6.05%)	17 (6.39%)	6 (5.26%)	
Blood type:				0.824
A	116 (30.53%)	82 (30.83%)	34 (29.82%)	
AB	51 (13.42%)	33 (12.41%)	18 (15.79%)	
B	106 (27.89%)	74 (27.82%)	32 (28.07%)	
O	107 (28.16%)	77 (28.95%)	30 (26.32%)	
Pathological diagnosis:				0.096
II	127 (33.42%)	97 (36.47%)	30 (26.32%)	
III	164 (43.16%)	113 (42.48%)	51 (44.74%)	
IV	89 (23.42%)	56 (21.05%)	33 (28.95%)	
Radiotherapy:				0.070
1	148 (38.95%)	112 (42.11%)	36 (31.58%)	
2	232 (61.05%)	154 (57.89%)	78 (68.42%)	
Chemotherap:				0.231
1	132 (34.74%)	98 (36.84%)	34 (29.82%)	
2	248 (65.26%)	168 (63.16%)	80 (70.18%)	
Smoke:				0.634
No	304 (80.00%)	215 (80.83%)	89 (78.07%)	
Yes	76 (20.00%)	51 (19.17%)	25 (21.93%)	
Drink:				0.697
No	303 (79.74%)	214 (80.45%)	89 (78.07%)	
Yes	77 (20.26%)	52 (19.55%)	25 (21.93%)	
IDH-1:				1.000
Negative	270 (71.05%)	189 (71.05%)	81 (71.05%)	
Positive	110 (28.95%)	77 (28.95%)	33 (28.95%)	
P53:				0.223
Negative	265 (69.74%)	191 (71.80%)	74 (64.91%)	
Positive	115 (30.26%)	75 (28.20%)	40 (35.09%)	
Ki67:				0.267
Negative	218 (57.37%)	158 (59.40%)	60 (52.63%)	
Positive	162 (42.63%)	108 (40.60%)	54 (47.37%)	
MGMT:				0.452
Methylated	57 (15.00%)	37 (13.91%)	20 (17.54%)	
Unmethylated	323 (85.00%)	229 (86.09%)	94 (82.46%)	
GFAP:				0.214
Negative	62 (16.32%)	48 (18.05%)	14 (12.28%)	
Positive	318 (83.68%)	218 (81.95%)	100 (87.72%)	
Tumor location:				0.353
left	190 (50.00%)	136 (51.13%)	54 (47.37%)	
right	177 (46.58%)	119 (44.74%)	58 (50.88%)	
right+left	13 (3.42%)	11 (4.14%)	2 (1.75%)	
NLR	2.23 [1.53;3.89]	2.26 [1.57;3.89]	2.08 [1.33;3.81]	0.242
PLR	135.53 [105.31;174.73]	136.65 [105.28;175.87]	131.07 [106.97;170.28]	0.649
MLR	0.29 [0.22;0.43]	0.30 [0.23;0.44]	0.28 [0.21;0.41]	0.297
PNI	48.83 [45.40;52.01]	48.67 [45.35;51.93]	49.65 [45.70;52.04]	0.233
Lasso score	-3.59 [-3.81;-3.36]	-3.56 [-3.78;-3.36]	-3.64 [-3.86;-3.34]	0.164

BMI, Body Mass Index; calculated as BMI = Weight (kg)/Height (m)²; KPS, Karnofsky Performance Status Score; Surgical removal: The extent of brain tumor resection. When the patient’s brain tumor removal volume is ≥95%, it is classified as “complete resection”; when the brain tumor removal volume is <95%, it is classified as “partial resection”; Midline shift: Indicates whether the midline of the brain exhibits a shift. “Yes” if a midline shift is present, and “No” if the midline is centered; Tumor texture: The texture of the resected tumor portion observed after surgery, primarily classified as soft, hard, or other; Radiotherapy: Indicates whether the patient received radiotherapy following surgery. “2” indicates the patient received radiotherapy, and “1” indicates the patient did not receive radiotherapy; Chemotherapy: Indicates whether the patient received chemotherapy following surgery. “2” indicates the patient received chemotherapy, and “1” indicates the patient did not receive chemotherapy; IDH-1: Isocitrate dehydrogenase 1, categorized as IDH-wild type (Negative) or IDH-mutant (Positive); P53: Tumor protein 53, classified according to its status as P53 wild type (Negative) or P53 mutant (Positive); Ki67: Ki67 expression status, categorized as “Negative (Ki67 <30%)” and “Positive (Ki67 ≥30%)”; MGMT: O-6-Methylguanine-DNA methyltransferase, classified according to its methylation status as “Methylated” or “Unmethylated”; GFAP, Glial fibrillary acidic protein. Expression of GFAP is designated as “Positive”, and absence of expression is designated as “Negative”; NLR, neutrophils/lymphocytes; PLR, platelets/lymphocytes; MLR, monocytes/lymphocytes; PNI, albumin (g/dL) + 5 × lymphocyte count (109/L); Lasso score: A blood index labeling, calculated as: Lasso score = (0.04738566 × NLR) + (-0.00196137 × PLR) + (-0.02408566 × MLR) + (-0.07038983 × PNI); P.overall: P-value. A P-value < 0.05 is considered statistically significant.

### 3.2 Lasso score

**Figure 2:**
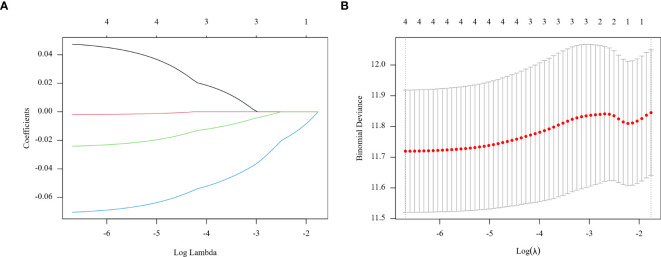
Regression coefficient path model **(A)** generated from the log (Lambda) values of the 4 features in the LASSO model. Parameter selection adjustment in the LASSO model 10 fold cross validation diagram **(B)**.

In the training group, prognostic markers NLR, PLR, MLR, and PNI were screened utilizing Lasso regression. Coefficients were derived at a minimal λ value of 0.00124078 (**Figure 2**). The Lasso score was then calculated from these four factors utilizing the following formula: (0.04738566 × NLR) + (-0.00196137 × PLR) + (-0.02408566 × MLR) + (-0.07038983 × PNI).

### 3.3 Multifactorial Cox proportional risk regression model

**Figure 3:**
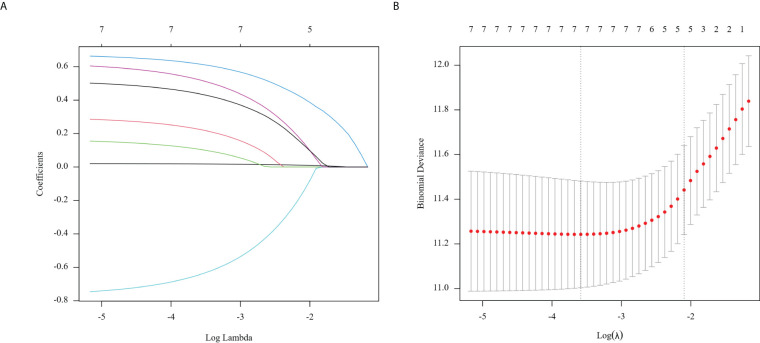
Regression coefficient path model **(A) **generated from the log (Lambda) values of the 7 features in the LASSO model. Parameter selection adjustment in the LASSO model 10 fold cross validation diagram **(B)**.

Clinicopathological characteristics of glioma patients in the training cohort, including age, sex, midline shift, tumor pathology type, IDH mutation status, and Lasso score, were evaluated utilizing univariate Cox proportional hazards regression. This analysis indicated that age, extent of resection, midline shift, tumor pathology type, chemotherapy administration, Ki-67 expression, and Lasso score were potential prognostic factors (all P < 0.05, **Table 2**). These seven factors were further analyzed utilizing Lasso regression, with the analytical process depicted in **Figure 3**. This identified age, tumor pathology type, chemotherapy administration, Ki-67 expression, and Lasso score as independent prognostic factors (P = 0.01, P < 0.001, P < 0.001, P < 0.001, and P = 0.007, respectively). Multivariable Cox proportional risks regression further confirmed the independent prognostic value of these five variables (**Table 3**).

**Table 2:** Results of univariate Cox proportional risk regression analysis of prognostic factors for glioma patients in the training cohort.

Variable	Level	HR(95%CI)	P
Age		1.037 (1.024, 1.050)	<0.001
Gender			
	Men	reference	
	Women	0.948 (0.681, 1.319)	0.750
BMI		0.970 (0.926, 1.016)	0.192
Kps		1.003 (0.991, 1.014)	0.668
Surgical removal			
	Complete resection	reference	
	Partial resection	1.456 (1.026, 2.064)	0.035
Midline shift			
	No	reference	
	Yes	1.471 (1.060, 2.041)	0.021
Tumor texture			
	Hard	reference	
	Other	1.290 (0.837, 1.987)	0.249
	Soft	0.999 (0.662, 1.508)	0.996
Tumor margin			
	Obscure	reference	
	Well-defined	0.749 (0.367, 1.531)	0.429
Blood type			
	A	reference	
	AB	0.921 (0.518, 1.639)	0.781
	B	1.191 (0.777, 1.828)	0.423
	O	1.296 (0.851, 1.974)	0.227
Pathological diagnosis			
	II	reference	
	III	3.025 (1.956, 4.676)	<0.001
	IV	4.947 (3.085, 7.933)	<0.001
Radiotherapy			
	1	reference	
	2	0.782 (0.563, 1.086)	0.142
Chemotherapy			
	1	reference	
	2	0.632 (0.454, 0.880)	0.007
Smoke			
	No	reference	
	Yes	1.035 (0.681, 1.573)	0.871
Drink			
	No	reference	
	Yes	1.227 (0.821, 1.835)	0.318
IDH-1			
	Negative	reference	
	Positive	0.731 (0.503, 1.062)	0.100
P53			
	Negative	reference	
	Positive	1.403 (0.987, 1.993)	0.059
Ki67			
	Negative	reference	
	Positive	2.001 (1.444, 2.774)	<0.001
MGMT			
	Methylated	reference	
	Unmethylated	0.925 (0.582, 1.469)	0.741
GFAP			
	Negative	reference	
	Positive	1.015 (0.670, 1.536)	0.945
Tumor location			
	left	reference	
	right	1.004 (0.719, 1.402)	0.980
	right+left	1.055 (0.458, 2.426)	0.900
Lasso score		2.780 (1.815, 4.258)	<0.001

Variable: predictor variable; Level: specific stratification corresponding to the predictor variable; CI, confidence interval; P, P-value, statistically significant at P < 0.05.

**Table 3:** Results of selection of variables for multivariate Cox proportional risk regression analysis.

Variable	Level	HR(95%CI)	P
Age		1.022 (1.008, 1.035)	0.001
Pathological diagnosis			
	II	reference	
	III	2.511 (1.607, 3.924)	<0.001
	IV	3.940 (2.395, 6.483)	<0.001
Chemotherapy			
	1	reference	
	2	0.470 (0.331, 0.667)	<0.001
Ki67			
	Negative	reference	
	Positive	1.838 (1.309, 2.581)	<0.001
Lasso score		1.825 (1.182, 2.819)	0.007

Variable: predictor variable; Level: specific stratification corresponding to the predictor variable; CI, confidence interval; P, P-value, statistically significant at P < 0.05.

The path model demonstrates regression coefficients calculated from the log(Lambda) values of the 7 features in the LASSO model (A). We implemented a 10-fold cross-validation model for parameter selection adjustment in the LASSO model (B). The least absolute shrinkage and selection operator (LASSO) cox regression algorithm helped identify significant features (with non-zero coefficients) from these parameters, while optimal parameter configurations were established through 10-fold cross-validation. This process determined the coefficients according to lambda values that corresponded to one standard error of distance from the minimum deviation of 0.12282066, finally filtering out five variables with non-zero coefficients.

### 3.4 Establishment of prediction model

**Figure 4:**
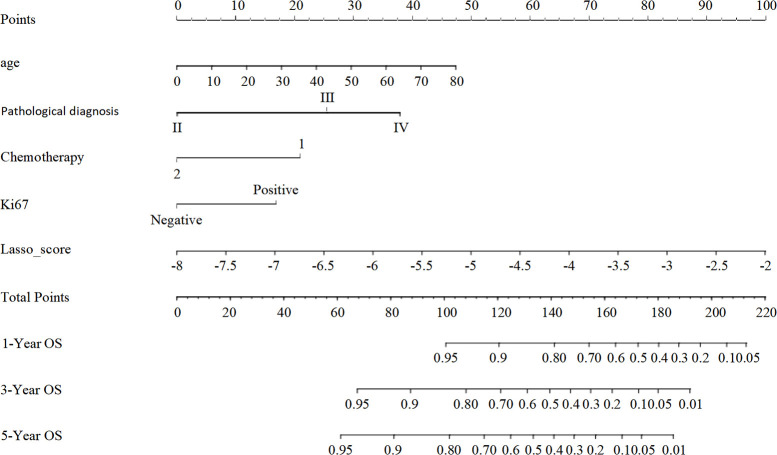
The nomogram model for predicting the prognosis of glioma patients at 1, 3 and 5 years after surgery. Individual scores corresponding to the different clinical variables taken in the nomogram diagrams were summed to correspond to different total scores, which corresponded to the OS of glioma patients at different time points at 1, 3, and 5 years postoperatively.

We developed a nomogram model drawing from the results of the multivariate Lasso Cox proportional risk regression analyses to estimate survival probabilities at 1-, 3-, and 5-years post-surgery for patients with glioma (**Figure 4**).

### 3.5 Validation and use of nomogram model

**Figure 5:**
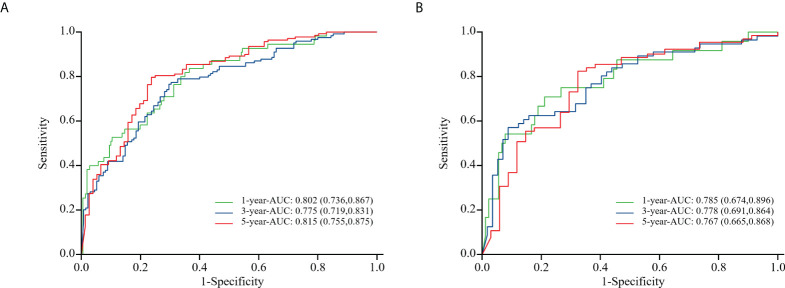
ROC curves of the training cohort **(A)** and the validation cohort **(B)** in nomogram model about glioma patients at 1, 3, and 5 years after surgery.

**Figure 6:**
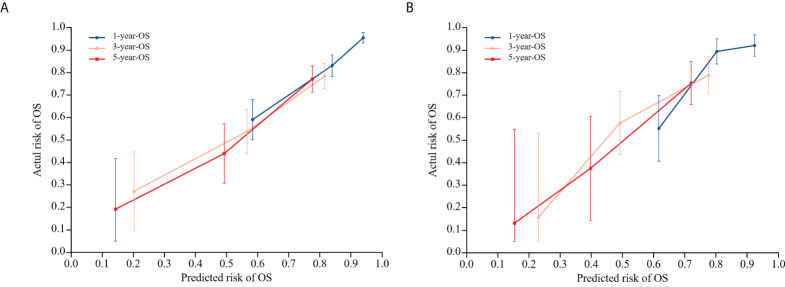
Calibration model of nomogram model on OS at 1, 3, and 5 years postoperatively in glioma patients in the training cohort **(A)**, and calibration model of column line model on OS at 1, 3, and 5 years postoperatively in glioma patients in the validation cohort **(B)**. 45° diagonal is the calibration model for the most optimal case (predicted probability = observed probability). The calibration model of the nomogram model regarding the OS of glioma patients at 1, 3, and 5 years after surgery is represented by the blue, orange, and red lines.

To evaluate the nomogram model’s effectiveness, we employed the C-index, ROC curves, and calibration model. For the training cohort, the nomogram model’s ROC curves yielded AUC values of 0.802, 0.755, and 0.815 at 1, 3, and 5 years after surgery, with a C index value of 0.742 (95% CI: 0.700-0.783)(**Figure 5A**). In the validation cohort, the ROC curves for the nomogram model at 1-, 3-, and 5-years post-surgery demonstrated AUC values of 0.785, 0.778, and 0.767, respectively, alongside a C index value of 0.734 (95% CI: 0.671-0.798)(**Figure 5B**). The calibration model analyzing 1-, 3-, and 5-year postoperative OS in glioma patients indicated strong correlation between predicted outcomes and actual observations across both training and validation cohorts (**Figure 6**). Our analysis also included calculating C-indexes (0.734 and 0.738) for the traditional clinical nomogram model in both cohorts, which we then compared with the C-indexes of our current nomogram model based on the inflammatory nutritional score. The inclusion of the Lasso score into traditional models failed to significantly improve OS predictions.

### 3.6 Therapeutic application

**Figure 7:**
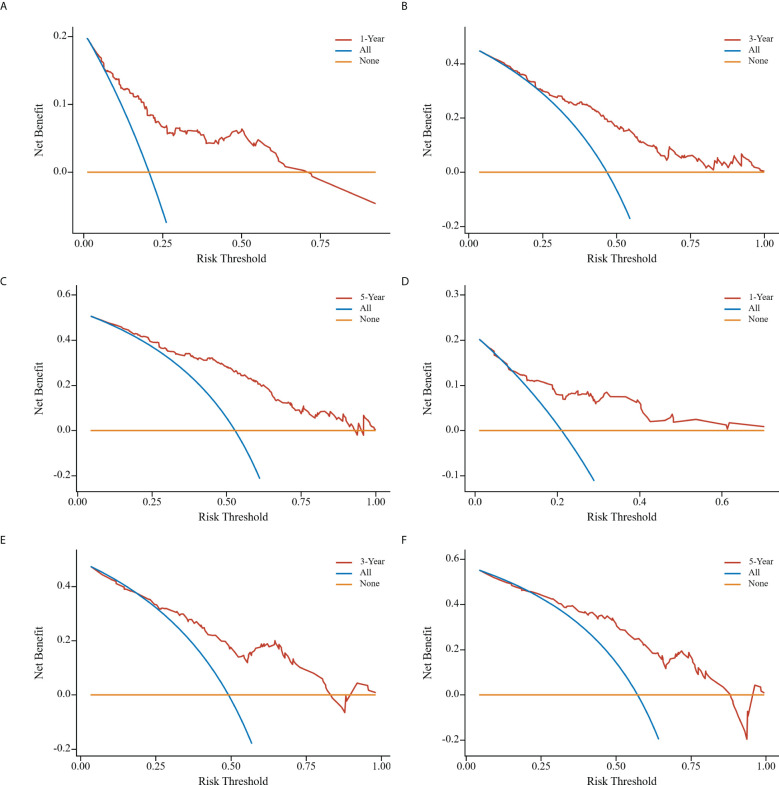
Clinical decision curve analyses of nomogram model in the training cohort **(A-C)**; clinical decision curve analyses of nomogram model in the validation cohort **(D-F)**. the x-axis represents the corresponding threshold probabilities, whereas the y-axis represents the net benefits. The solid orange line represents the gain curve when all patients do not receive treatment, the solid blue line represents the net clinical gain curve when all patients are treated, and the solid red line represents the net clinical gain curve when patients in each of the prediction thresholds of the nomogram diagram are treated.

Clinical DCA was employed to evaluate the predictive model’s clinical utility. The threshold probabilities of the nomogram model in the training cohort at 1, 3, and 5 years after surgery were 0.08~0.74, 0.25~0.80, and 0.08~0.89, respectively. Corresponding probabilities in the validation cohort were 0.13~0.60, 0.28~0.81, and 0.25~0.88, respectively (**Figure 7**).

### 3.7 Risk group survival curves

**Figure 8:**
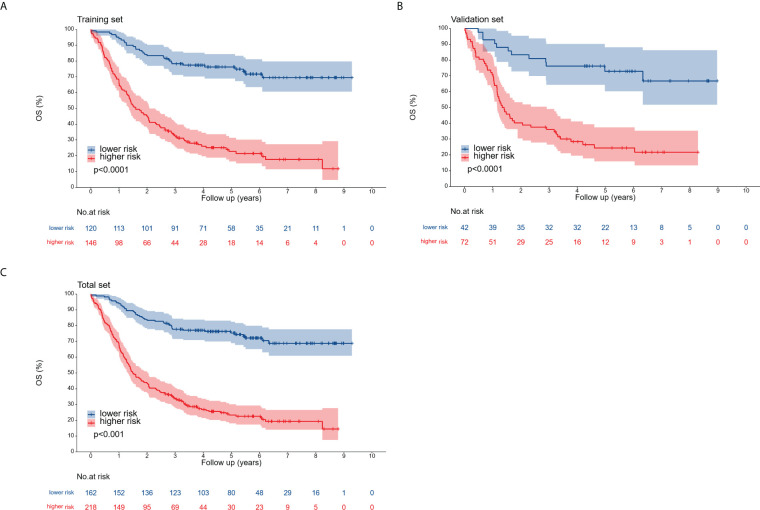
Overall survival curves for the training cohort **(A)**, validation cohort **(B)**, and complete cohort **(C)**, categorized into low-risk and high-risk groups according to the optimal cutoff point.

Patients across the entire cohort, as well as in the training and validation cohorts, were stratified into high-risk and low-risk groups. In the training cohort, 1-, 3-, and 5-year postoperative survival rates for the high-risk group were 67.1%, 32.7%, and 22.7%, respectively, while the low-risk group demonstrated corresponding rates of 94.2%, 78.2%, and 76.2%. While patients classified as high-risk experienced a less favorable prognosis compared to the low-risk group, this difference was not significant. In the validation cohort, the 1-, 3-, and 5-year postoperative survival rates were 70.8%, 36.1%, and 24.4% for the high-risk group and 92.9%, 76.2%, and 72.9% for the low-risk group. For the entire cohort, these rates were 68.3%, 33.8%, and 23.3% (high-risk) and 93.8%, 77.7%, and 75.3% (low-risk), respectively. The outcomes observed in the training group aligned with those of the validation and entire cohorts (**Figure 8**).

## 4 Discussion

Gliomas constitute approximately 80% of primary malignant brain tumors (12). Despite the availability of various treatment modalities, including surgery, chemotherapy, radiotherapy, and targeted therapy, long-term survival for patients with glioma remains limited. While pathologic grading is crucial for guiding treatment decisions and prognostication, patients with identical pathologic grades can exhibit significant difference in survival outcomes in clinical settings. This suggests that current grading systems may not accurately reflect the prognosis for patients with glioma. Importantly, this study indicated a correlation between tumor development, prognosis, and the inflammatory response. Therefore, we sought to develop a nomogram based on hematological and clinical tumor characteristics to facilitate individualized treatment planning and follow-up care. The optimal markers for predicting glioma prognosis were identified by considering relevant nutritional and inflammatory indices in peripheral blood, alongside pathologic grade and treatment approaches.

In 1881, the German physician Virchow pioneered the analysis into the relationship between tumors and inflammation, a topic that has since been the subject of extensive research (13). A growing body of evidence indicates that inflammation plays a crucial role in tumor invasion, metastasis, and recurrence. For instance, inflammation has been implicated in disrupting genomic integrity and facilitating tumor growth by increasing DNA damage in tissues and compromising DNA repair mechanisms (14). Peripheral blood components, including neutrophils, lymphocytes, and platelets, are believed to be intimately involved in tumor development. Neutrophils can contribute to tumor growth through the release of granule proteins such as matrix metalloproteinase-9, which degrades the extracellular matrix; vascular endothelial growth factor (VEGF), which stimulates tumor angiogenesis; and arginase-1, which suppresses T-cell activation and induces immunosuppression (15). Cytotoxic T lymphocytes, the primary lymphocytes with anti-tumor activity, exert their effects by releasing cytotoxic particles such as perforin and granzyme, leading to the lysis and destruction of tumor cells (16). Platelets have also been demonstrated to facilitate the metastasis of tumor cells to distant sites by shielding circulating tumor cells from immune surveillance and the high shear stress of the circulatory system, as well as by secreting ATP and promoting epithelial–mesenchymal transition in tumor cells (17). In addition, studies have indicated an association between high albumin levels in tumor patients, decreased immune function, and increased mortality (18). Further research on tumor prognostic variables, including NLR, PLR, MLR, and PNI, has incorporated several inflammatory indices based on the aforementioned blood component combinations. Qi et al. reported an association between high NLR and adverse outcomes in 214 patients with low-grade gliomas (19), while Wang et al. identified NLR and PNI as independent prognostic factors for OS in glioma patients (20). Another study indicated that high PLR and MLR values correlated with poorer prognoses in tumor patients (21); however, Yan et al. observed that, while PLR and MLR levels differed among groups of glioma patients, these markers did not independently predict OS (10). The prognostic value of these inflammatory and nutritional markers in glioma therefore remains a subject of debate, with most research focusing on the predictive value of individual inflammatory or nutritional indicators. Considering the potential for the mechanisms underlying these various inflammatory and nutritional markers to differentially affect glioma progression, we conducted an analysis incorporating a combination of these factors.

A Lasso Cox regression algorithm, which identifies correlations between predictor variables and outcomes by shrinking the regression coefficients of various variables, was employed in this study. This approach to variable selection enhances the stability of the resulting model compared with the statistical modeling approaches employed in other studies. Because individual risk factors have limited power for predicting tumor prognosis due to tumor heterogeneity, a nomogram model was developed to integrate multiple risk factors, thereby exploiting the combined predictive potential of these factors to improve prognostic accuracy for tumor outcomes. Specifically, age, pathological grade, chemotherapy, Ki-67, and Lasso score were selected as variables for constructing the nomogram model. This model derived C-indexes of 0.742 and 0.734 in the training and validation cohorts, respectively. Moreover, among the preoperative prognostic factors, the Lasso score is the dominant factor in the nomogram, accounting for the highest score, indicating that the Lasso score may be a highly effective preoperative prognostic factor. Moreover, a traditional nomogram model was constructed to assess the specific contribution of the Lasso score to the prediction model’s performance. This traditional model produced C-indexes of 0.734 and 0.738 in the training and validation cohorts, respectively.

A comprehensive evaluation of the performance and clinical utility of the Lasso score-based nomogram model was performed utilizing the validation cohort. Validation results, based on the C-index and calibration curves, demonstrated that the Lasso score-based nomogram model exhibits stable and robust predictive effectiveness in the validation cohort dataset. The clinical utility of the Lasso score-based nomogram model was also rigorously verified with DCA. DCA, which leverages the threshold probability of interpreting clinical outcomes to derive the net clinical benefit, indicated a high net clinical benefit associated with the Lasso score-based nomogram (considering 1-, 3-, and 5-year postoperative values for the validation cohort). The corresponding 1-, 3-, and 5-year threshold probabilities were 0.13~0.60, 0.28~0.81, and 0.25~0.81, respectively.

In addition, the nomogram model constructed utilizing the Lasso score in this study offers a mechanism for stratifying patients according to their prognostic risks. This risk stratification capability has been verified across all cohorts, including the independent validation cohort. The identification of high-risk patients through this stratification allows for closer follow-up and monitoring to detect any disease progression to allow for subsequent individualized treatment strategies.

Compared with models constructed using single or a few factors, our study integrates multiple hematological and clinical factors to comprehensively evaluate patients’ prognosis from multiple dimensions, enabling us to provide more abundant and accurate prognostic information. In contrast to other nomogram models based on gene - level, specific protein and other indicators (22–24), our study constructs the nomogram model using data that can be obtained through routine clinical means. It does not rely on complex gene - testing techniques, expensive high - end equipment, or special experimental conditions, thus improving the clinical practicability and feasibility of the model.

Notwithstanding its contributions, this study is not without certain limitations. First, despite the significant scale of the study population (N=380), its retrospective design, confined to a single institution, introduces the possibility of selection bias. Therefore, further studies including larger study populations and incorporating data derived from multicenter prospective analyses are necessary to verify the present findings. Second, peripheral blood markers associated with inflammatory response and nutritional status are also subject to the effect of other factors. To our knowledge, tumors and infectious diseases exhibit similarities in their respective changes of hematological markers (25). Increased NLR values have been documented in bacterial and fungal infections (26, 27). Moreover, NLR is regarded as a reliable hematological prognostic indicator in sepsis studies (28). Similarly, changes in NLR values have been observed in patients diagnosed with glioblastoma (GBM), where low preoperative NLR values have been associated with a more favorable patient prognosis (29). Therefore, patients presenting with infectious lesions were excluded from the inclusion criteria of this study. Further, the systemic inflammatory state is of crucial importance to the prognosis of patients with tumors. Specifically, it has been reported that patients diagnosed with human immunodeficiency virus (HIV)-associated colorectal cancer (CRC) demonstrate a worse prognosis compared to patients diagnosed with non-HIV-associated CRC (30). This may be attributed to declined serum albumin and lymphocyte counts. Nevertheless, despite these limitations, we maintain that this study offers novel insights and guidance regarding the predictive value of inflammatory and nutritional markers in relation to the prognosis of glioma patients.

In conclusion, while the Lasso score-based nomogram model did not significantly enhance the performance of the traditional nomogram model, it exhibited robust performance in predicting the prognosis of glioma patients in both the training and validation cohorts. Considering their characteristics as non-invasive, low-cost, and repeatable prognostic markers, the nutritional and inflammatory indicators necessary for the construction of the Lasso score are readily obtainable through routine hematological examinations. Therefore, the Lasso score, derived from the nomogram model, facilitates individualized prognostic predictions and demonstrates promising clinical potential for application in the management of patients with glioma.

## Data Availability

The raw data supporting the conclusions of this article will be made available by the authors, without undue reservation.
